# {4,4′,6,6′-Tetra­chloro-2,2′-[(spiro­[4.4]nonane-1,6-di­yl)bis­(nitrilo­methyl­idyne)]diphenolato-κ^4^ *O*,*N*,*N*′,*O*′}nickel(II)

**DOI:** 10.1107/S1600536808013792

**Published:** 2008-06-13

**Authors:** Fan Ni, Zhi-Qing Wu, Lei Liang, Xiang-Ge Zhou

**Affiliations:** a Institute of Homogeneous Catalysis, Department of Chemistry, Sichuan University, Chengdu 610064, People’s Republic of China

## Abstract

The title compound, [Ni(C_23_H_20_Cl_4_N_2_O_2_)], has an Ni^II^ ion in a square-planar coordination formed by two imine N and two phenolato O atoms.

## Related literature

For related literature, see: Gaetani Manfredotti *et al.* (1983 ▶), de Castro *et al.* (2001 ▶); Lutz (2003 ▶); Hoshina *et al.* (2000 ▶); Gosden *et al.* (1978 ▶, 1981 ▶); Healy & Pletcher (1980 ▶); Dahm & Peters (1996 ▶).
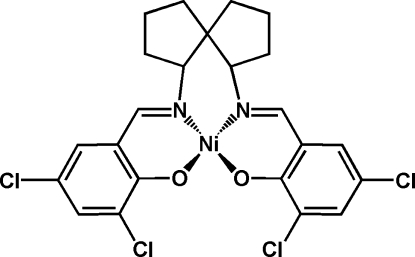


## Experimental

### 

#### Crystal data

[Ni(C_23_H_20_Cl_4_N_2_O_2_)]
*M* *_r_* = 556.92
Monoclinic, 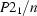

*a* = 13.344 (2) Å
*b* = 12.073 (2) Å
*c* = 14.081 (2) Å
β = 97.181 (3)°
*V* = 2250.6 (6) Å^3^
*Z* = 4
Mo *K*α radiation
μ = 1.36 mm^−1^
*T* = 294 (2) K
0.22 × 0.20 × 0.12 mm

#### Data collection

Bruker SMART CCD area-detector diffractometer
Absorption correction: multi-scan (*SADABS*; Sheldrick, 1996 ▶) *T*_min_ = 0.761, *T*_max_ = 1.000 (expected range = 0.646–0.849)
20414 measured reflections
5196 independent reflections
3731 reflections with *I* > 2σ(*I*)
*R*_int_ = 0.055

#### Refinement

*R*[*F* ^2^ > 2σ(*F* ^2^)] = 0.038
*wR*(*F* ^2^) = 0.113
*S* = 1.01
5196 reflections
289 parameters
H-atom parameters constrained
Δρ_max_ = 0.55 e Å^−3^
Δρ_min_ = −0.40 e Å^−3^

### 

Data collection: *SMART* (Bruker, 1997 ▶); cell refinement: *SAINT* (Bruker, 1997 ▶); data reduction: *SAINT*; program(s) used to solve structure: *SHELXS97* (Sheldrick, 2008 ▶); program(s) used to refine structure: *SHELXL97* (Sheldrick, 2008 ▶); molecular graphics: *SHELXTL* (Sheldrick, 2008 ▶); software used to prepare material for publication: *SHELXTL*.

## Supplementary Material

Crystal structure: contains datablocks I, global. DOI: 10.1107/S1600536808013792/im2062sup1.cif

Structure factors: contains datablocks I. DOI: 10.1107/S1600536808013792/im2062Isup2.hkl

Additional supplementary materials:  crystallographic information; 3D view; checkCIF report

## Figures and Tables

**Fig. 1.**
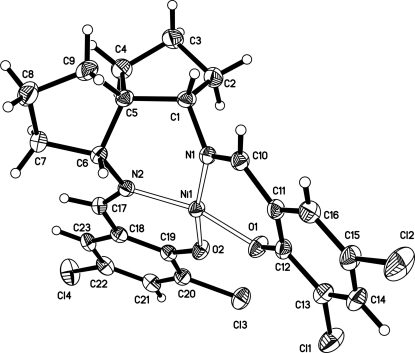
A view of complex [Ni(C23H20Cl4N2O2)], with displacement ellipsoids drawn at the 30% probability level.

## supplementary crystallographic information

### Comment

Nickel(II) complexes with N_2_O_2_ Schiff base ligands derived from salicylaldehyde have long been used as homogenous catalysts (Gosden *et al.*, 1978, 1981; Healy & Pletcher, 1980). More recently, the preparation of metal-salen based modified electrodes by oxidative electropolymerization of the metal complexes prompted their use in heterogenous electrocatalysis (Dahm & Peters, 1996). Work in our laboratory has attempted to introduce spiro[4.4]nonane-1,6-diamine as backbone into the salen system and investigate its coordination feature.

The crystal structure of the title compound 1 is shown in Fig. 1, while bond lengths and angles are listed in the supplementary material. As shown in Fig.1, the mononuclear Ni^II^ ion is tetra-coordinated, showing a nearly perfectly square-planar coordination mode. The planes Ni1—N1—C10—C11—C12—O1 and Ni1—Ni2—C17—C18—C19—O2 are not coplanar due to the steric pressure of the spirocyclic ligand.

The O—Ni—O, N—Ni—N and N—Ni—O angles correspond very well with the familiar Ni-salen complexes based on 1,2-ethanediamine (Gaetani Manfredotti *et al.* 1983, Lutz, 2003), 1,2-cyclohexanediamine (Castro *et al.* 2001), and 1,2- diphenyl-1,2-ethanediamine (Hoshina *et al.* 2000). de Castro *et al.* found that the coordination geometry usually is tetrahedrally distorted the more the substituents in the imine bridge are bulkier or if the substitution is asymmetric. Here we attribute the intensive distortion to the spiro frame which reinforces the asymmetry.

A comparison with the three analogous nickel complexes above indicates that, in the present compound, both the Ni—O bonding distances [1.848 (2) / 1.846 (2), respectively] are in good agreement with those observed in similar Schiff base Ni complexes whereas the Ni—N bonding distances [1.892 (2) / 1.884 (2) Å, respectively] are slightly longer [reported values range from 1.843 (2) to 1.855 (2) Å].

### Experimental

The title complex, [*N*,*N*'-Bis(3,5-dichloro-salicylidene)- spiro[4.4]nonane-1,6-diaminato]-nickel(II), was prepared by the reaction of a hot methanolic solution (30 mL) of nickel(II) acetate tetrahydrate (0.249 g, 1 mmol) with the Schiff base ligand *N*,*N*'-Bis(3,5-dichloro-salicylidene)-spiro[4.4]nonane-1,6-diamine (0.500 g, 1 mmol). The resulting green precipitate was collected by filtration and washed with methanol and ether (yield 38%). Dark green crystals of 1 were grown by slow diffusion of ether into a solution of 1 in dichloromethane.

### Refinement

All hydrogen atoms of the complex were positioned geometrically and refined using a riding model, with C—H = 0.93 Å (aromatic) and 0.98 Å (methylene) with *U*_iso_(H) =1.2Ueq (C).

### Crystal data

**Table:**

[Ni(C_23_H_20_Cl_4_N_2_O_2_)]	*F*_000_ = 1136
*M**_r_* = 556.92	*D*_x_ = 1.644 Mg m^−^^3^
Monoclinic, *P*2_1_/*n*	Mo *K*α radiation λ = 0.71073 Å
Hall symbol: -P 2yn	Cell parameters from 10509 reflections
*a* = 13.344 (2) Å	θ = 1–27.5º
*b* = 12.073 (2) Å	µ = 1.36 mm^−^^1^
*c* = 14.081 (2) Å	*T* = 294 (2) K
β = 97.181 (3)º	Prism, black
*V* = 2250.6 (6) Å^3^	0.22 × 0.20 × 0.12 mm
*Z* = 4	

### Data collection

**Table:**

Bruker SMART CCD area-detector diffractometer	5196 independent reflections
Radiation source: fine-focus sealed tube	3731 reflections with *I* > 2σ(*I*)
Monochromator: graphite	*R*_int_ = 0.055
*T* = 294(2) K	θ_max_ = 27.6º
φ and ω scans	θ_min_ = 2.0º
Absorption correction: multi-scan(SADABS; Sheldrick, 1996)	*h* = −17→17
*T*_min_ = 0.761, *T*_max_ = 1.000	*k* = −15→15
20414 measured reflections	*l* = −18→18

### Refinement

**Table:**

Refinement on *F*^2^	Secondary atom site location: difference Fourier map
Least-squares matrix: full	Hydrogen site location: inferred from neighbouring sites
*R*[*F*^2^ > 2σ(*F*^2^)] = 0.039	H-atom parameters constrained
*wR*(*F*^2^) = 0.113	*w* = 1/[σ^2^(*F*_o_^2^) + (0.065*P*)^2^] where *P* = (*F*_o_^2^ + 2*F*_c_^2^)/3
*S* = 1.01	(Δ/σ)_max_ < 0.001
5196 reflections	Δρ_max_ = 0.55 e Å^−^^3^
289 parameters	Δρ_min_ = −0.39 e Å^−^^3^
Primary atom site location: structure-invariant direct methods	Extinction correction: none

### Special details

**Table:**

Geometry. All e.s.d.'s (except the e.s.d. in the dihedral angle between two l.s. planes) are estimated using the full covariance matrix. The cell e.s.d.'s are taken into account individually in the estimation of e.s.d.'s in distances, angles and torsion angles; correlations between e.s.d.'s in cell parameters are only used when they are defined by crystal symmetry. An approximate (isotropic) treatment of cell e.s.d.'s is used for estimating e.s.d.'s involving l.s. planes.
Refinement. Refinement of *F*^2^ against ALL reflections. The weighted *R*-factor *wR* and goodness of fit *S* are based on *F*^2^, conventional *R*-factors *R* are based on *F*, with *F* set to zero for negative *F*^2^. The threshold expression of *F*^2^ > σ(*F*^2^) is used only for calculating *R*-factors(gt) *etc*. and is not relevant to the choice of reflections for refinement. *R*-factors based on *F*^2^ are statistically about twice as large as those based on *F*, and *R*- factors based on ALL data will be even larger.

### Fractional atomic coordinates and isotropic or equivalent isotropic displacement parameters (Å^2^)

**Table:**

	*x*	*y*	*z*	*U*_iso_*/*U*_eq_	
Ni1	0.42863 (2)	0.43913 (3)	0.65679 (2)	0.02559 (11)	
Cl1	0.10295 (6)	0.32950 (8)	0.53445 (7)	0.0554 (2)	
Cl2	−0.05657 (6)	0.63477 (9)	0.75125 (8)	0.0661 (3)	
Cl3	0.41217 (6)	0.10984 (6)	0.47916 (6)	0.0453 (2)	
Cl4	0.78080 (7)	0.18824 (7)	0.37495 (7)	0.0568 (2)	
O1	0.29376 (14)	0.39968 (17)	0.63812 (14)	0.0377 (5)	
O2	0.43951 (13)	0.32517 (15)	0.57124 (13)	0.0327 (4)	
N1	0.41147 (15)	0.52722 (18)	0.76468 (15)	0.0276 (5)	
N2	0.55652 (15)	0.49892 (17)	0.64347 (15)	0.0267 (5)	
C1	0.49859 (19)	0.5405 (2)	0.84037 (18)	0.0306 (6)	
H1A	0.4785	0.5892	0.8904	0.037*	
C2	0.5314 (2)	0.4293 (3)	0.8853 (2)	0.0416 (7)	
H2A	0.5236	0.3704	0.8382	0.050*	
H2B	0.4928	0.4110	0.9372	0.050*	
C3	0.6429 (2)	0.4480 (3)	0.9227 (2)	0.0441 (8)	
H3A	0.6505	0.4916	0.9810	0.053*	
H3B	0.6786	0.3784	0.9344	0.053*	
C4	0.6798 (2)	0.5111 (3)	0.8402 (2)	0.0410 (7)	
H4A	0.6944	0.4605	0.7902	0.049*	
H4B	0.7405	0.5526	0.8622	0.049*	
C5	0.59263 (19)	0.5905 (2)	0.80250 (18)	0.0285 (6)	
C6	0.58053 (19)	0.6042 (2)	0.69372 (18)	0.0274 (5)	
H6A	0.5229	0.6537	0.6767	0.033*	
C7	0.6754 (2)	0.6688 (2)	0.6779 (2)	0.0366 (6)	
H7A	0.6677	0.7045	0.6157	0.044*	
H7B	0.7343	0.6210	0.6833	0.044*	
C8	0.6830 (2)	0.7548 (3)	0.7596 (2)	0.0480 (8)	
H8A	0.6586	0.8264	0.7355	0.058*	
H8B	0.7525	0.7627	0.7886	0.058*	
C9	0.6165 (2)	0.7099 (3)	0.8331 (2)	0.0431 (7)	
H9A	0.6526	0.7124	0.8973	0.052*	
H9B	0.5549	0.7529	0.8317	0.052*	
C10	0.32576 (19)	0.5653 (2)	0.78429 (19)	0.0295 (6)	
H10A	0.3273	0.6123	0.8368	0.035*	
C11	0.22883 (19)	0.5419 (2)	0.73250 (19)	0.0302 (6)	
C12	0.21902 (19)	0.4569 (2)	0.66395 (19)	0.0304 (6)	
C13	0.1189 (2)	0.4320 (2)	0.6213 (2)	0.0367 (6)	
C14	0.0362 (2)	0.4869 (3)	0.6479 (2)	0.0421 (7)	
H14A	−0.0285	0.4692	0.6193	0.051*	
C15	0.0498 (2)	0.5684 (3)	0.7173 (2)	0.0397 (7)	
C16	0.1439 (2)	0.5968 (2)	0.7601 (2)	0.0354 (6)	
H16A	0.1517	0.6517	0.8068	0.043*	
C17	0.6168 (2)	0.4638 (2)	0.58462 (18)	0.0290 (6)	
H17A	0.6717	0.5085	0.5757	0.035*	
C18	0.6061 (2)	0.3617 (2)	0.53205 (18)	0.0280 (6)	
C19	0.51800 (19)	0.2961 (2)	0.53137 (18)	0.0278 (5)	
C20	0.5172 (2)	0.1946 (2)	0.4804 (2)	0.0321 (6)	
C21	0.5954 (2)	0.1621 (2)	0.4321 (2)	0.0355 (6)	
H21A	0.5921	0.0955	0.3988	0.043*	
C22	0.6801 (2)	0.2305 (2)	0.4335 (2)	0.0359 (6)	
C23	0.6860 (2)	0.3287 (2)	0.48250 (19)	0.0324 (6)	
H23A	0.7428	0.3734	0.4829	0.039*	

### Atomic displacement parameters (Å^2^)

**Table:**

	*U*^11^	*U*^22^	*U*^33^	*U*^12^	*U*^13^	*U*^23^
Ni1	0.02081 (18)	0.02942 (19)	0.02673 (19)	0.00060 (13)	0.00379 (13)	−0.00226 (14)
Cl1	0.0407 (4)	0.0607 (6)	0.0629 (5)	−0.0066 (4)	−0.0008 (4)	−0.0221 (4)
Cl2	0.0264 (4)	0.0838 (7)	0.0887 (7)	0.0125 (4)	0.0095 (4)	−0.0225 (6)
Cl3	0.0416 (4)	0.0319 (4)	0.0620 (5)	−0.0045 (3)	0.0044 (4)	−0.0038 (3)
Cl4	0.0631 (5)	0.0467 (5)	0.0688 (6)	0.0084 (4)	0.0402 (5)	−0.0077 (4)
O1	0.0227 (10)	0.0430 (11)	0.0477 (12)	−0.0003 (8)	0.0057 (8)	−0.0123 (9)
O2	0.0251 (9)	0.0325 (10)	0.0416 (11)	−0.0006 (8)	0.0077 (8)	−0.0089 (8)
N1	0.0216 (11)	0.0342 (12)	0.0274 (11)	−0.0005 (9)	0.0044 (9)	0.0002 (9)
N2	0.0244 (11)	0.0294 (12)	0.0262 (11)	0.0014 (9)	0.0030 (9)	0.0002 (9)
C1	0.0239 (13)	0.0420 (16)	0.0258 (13)	0.0021 (11)	0.0023 (10)	−0.0030 (11)
C2	0.0359 (16)	0.0485 (18)	0.0393 (17)	−0.0031 (13)	0.0006 (13)	0.0122 (14)
C3	0.0333 (16)	0.059 (2)	0.0385 (17)	0.0051 (14)	−0.0034 (13)	0.0139 (15)
C4	0.0268 (15)	0.0538 (19)	0.0418 (17)	0.0066 (13)	0.0012 (12)	0.0058 (15)
C5	0.0217 (12)	0.0345 (15)	0.0287 (14)	0.0004 (10)	0.0003 (10)	−0.0017 (11)
C6	0.0236 (13)	0.0274 (13)	0.0308 (14)	−0.0008 (10)	0.0020 (10)	−0.0008 (11)
C7	0.0332 (15)	0.0364 (16)	0.0406 (16)	−0.0072 (12)	0.0065 (12)	0.0014 (13)
C8	0.0472 (19)	0.0435 (18)	0.054 (2)	−0.0134 (15)	0.0088 (15)	−0.0082 (15)
C9	0.0445 (18)	0.0451 (18)	0.0392 (17)	−0.0112 (14)	0.0030 (13)	−0.0129 (14)
C10	0.0271 (13)	0.0315 (14)	0.0306 (14)	0.0006 (11)	0.0059 (11)	−0.0010 (11)
C11	0.0232 (13)	0.0360 (15)	0.0323 (14)	0.0008 (11)	0.0067 (11)	0.0031 (11)
C12	0.0239 (13)	0.0332 (15)	0.0350 (15)	−0.0011 (11)	0.0069 (11)	0.0024 (12)
C13	0.0312 (15)	0.0387 (16)	0.0393 (16)	−0.0040 (12)	0.0007 (12)	−0.0018 (13)
C14	0.0224 (14)	0.0531 (19)	0.0500 (18)	−0.0021 (13)	0.0015 (12)	−0.0004 (15)
C15	0.0233 (14)	0.0472 (18)	0.0493 (18)	0.0057 (12)	0.0077 (12)	0.0011 (14)
C16	0.0306 (15)	0.0394 (16)	0.0368 (16)	0.0069 (12)	0.0067 (12)	0.0010 (12)
C17	0.0269 (13)	0.0313 (14)	0.0292 (14)	−0.0031 (11)	0.0055 (11)	0.0014 (11)
C18	0.0301 (14)	0.0283 (14)	0.0259 (13)	0.0054 (11)	0.0043 (11)	0.0020 (11)
C19	0.0284 (13)	0.0278 (13)	0.0275 (13)	0.0039 (11)	0.0042 (11)	0.0019 (11)
C20	0.0324 (14)	0.0295 (14)	0.0339 (15)	0.0015 (11)	0.0017 (11)	0.0028 (11)
C21	0.0465 (17)	0.0272 (14)	0.0338 (15)	0.0060 (13)	0.0093 (13)	0.0000 (12)
C22	0.0398 (16)	0.0356 (16)	0.0348 (15)	0.0108 (13)	0.0146 (12)	0.0024 (12)
C23	0.0325 (15)	0.0343 (15)	0.0321 (15)	0.0014 (12)	0.0109 (12)	0.0052 (12)

### Geometric parameters (Å, °)

**Table:**

Ni1—O2	1.8463 (18)	C6—H6A	0.9800
Ni1—O1	1.8484 (19)	C7—C8	1.544 (4)
Ni1—N2	1.884 (2)	C7—H7A	0.9700
Ni1—N1	1.892 (2)	C7—H7B	0.9700
Cl1—C13	1.734 (3)	C8—C9	1.544 (4)
Cl2—C15	1.747 (3)	C8—H8A	0.9700
Cl3—C20	1.733 (3)	C8—H8B	0.9700
Cl4—C22	1.739 (3)	C9—H9A	0.9700
O1—C12	1.302 (3)	C9—H9B	0.9700
O2—C19	1.297 (3)	C10—C11	1.432 (4)
N1—C10	1.294 (3)	C10—H10A	0.9300
N1—C1	1.484 (3)	C11—C12	1.403 (4)
N2—C17	1.296 (3)	C11—C16	1.409 (4)
N2—C6	1.470 (3)	C12—C13	1.426 (4)
C1—C2	1.525 (4)	C13—C14	1.379 (4)
C1—C5	1.546 (4)	C14—C15	1.383 (4)
C1—H1A	0.9800	C14—H14A	0.9300
C2—C3	1.532 (4)	C15—C16	1.366 (4)
C2—H2A	0.9700	C16—H16A	0.9300
C2—H2B	0.9700	C17—C18	1.436 (4)
C3—C4	1.522 (4)	C17—H17A	0.9300
C3—H3A	0.9700	C18—C23	1.403 (4)
C3—H3B	0.9700	C18—C19	1.416 (4)
C4—C5	1.548 (4)	C19—C20	1.420 (4)
C4—H4A	0.9700	C20—C21	1.372 (4)
C4—H4B	0.9700	C21—C22	1.397 (4)
C5—C9	1.527 (4)	C21—H21A	0.9300
C5—C6	1.529 (4)	C22—C23	1.368 (4)
C6—C7	1.526 (4)	C23—H23A	0.9300
			
O2—Ni1—O1	82.52 (8)	H7A—C7—H7B	109.2
O2—Ni1—N2	94.27 (8)	C7—C8—C9	105.9 (2)
O1—Ni1—N2	164.28 (9)	C7—C8—H8A	110.6
O2—Ni1—N1	165.98 (9)	C9—C8—H8A	110.6
O1—Ni1—N1	92.62 (9)	C7—C8—H8B	110.6
N2—Ni1—N1	93.82 (9)	C9—C8—H8B	110.6
C12—O1—Ni1	126.14 (18)	H8A—C8—H8B	108.7
C19—O2—Ni1	128.15 (17)	C5—C9—C8	105.0 (2)
C10—N1—C1	116.2 (2)	C5—C9—H9A	110.7
C10—N1—Ni1	124.76 (18)	C8—C9—H9A	110.7
C1—N1—Ni1	118.34 (16)	C5—C9—H9B	110.7
C17—N2—C6	118.3 (2)	C8—C9—H9B	110.7
C17—N2—Ni1	125.51 (19)	H9A—C9—H9B	108.8
C6—N2—Ni1	115.40 (15)	N1—C10—C11	126.0 (3)
N1—C1—C2	111.2 (2)	N1—C10—H10A	117.0
N1—C1—C5	113.0 (2)	C11—C10—H10A	117.0
C2—C1—C5	106.5 (2)	C12—C11—C16	121.6 (2)
N1—C1—H1A	108.7	C12—C11—C10	119.7 (2)
C2—C1—H1A	108.7	C16—C11—C10	118.2 (3)
C5—C1—H1A	108.7	O1—C12—C11	124.8 (2)
C1—C2—C3	103.2 (2)	O1—C12—C13	118.7 (2)
C1—C2—H2A	111.1	C11—C12—C13	116.5 (2)
C3—C2—H2A	111.1	C14—C13—C12	121.6 (3)
C1—C2—H2B	111.1	C14—C13—Cl1	120.2 (2)
C3—C2—H2B	111.1	C12—C13—Cl1	118.3 (2)
H2A—C2—H2B	109.1	C13—C14—C15	119.7 (3)
C4—C3—C2	101.8 (2)	C13—C14—H14A	120.2
C4—C3—H3A	111.4	C15—C14—H14A	120.2
C2—C3—H3A	111.4	C16—C15—C14	121.5 (3)
C4—C3—H3B	111.4	C16—C15—Cl2	119.8 (2)
C2—C3—H3B	111.4	C14—C15—Cl2	118.7 (2)
H3A—C3—H3B	109.3	C15—C16—C11	119.1 (3)
C3—C4—C5	105.7 (2)	C15—C16—H16A	120.4
C3—C4—H4A	110.6	C11—C16—H16A	120.4
C5—C4—H4A	110.6	N2—C17—C18	125.5 (2)
C3—C4—H4B	110.6	N2—C17—H17A	117.3
C5—C4—H4B	110.6	C18—C17—H17A	117.3
H4A—C4—H4B	108.7	C23—C18—C19	121.3 (2)
C9—C5—C6	99.9 (2)	C23—C18—C17	117.9 (2)
C9—C5—C1	114.9 (2)	C19—C18—C17	120.9 (2)
C6—C5—C1	113.5 (2)	O2—C19—C18	124.0 (2)
C9—C5—C4	111.5 (2)	O2—C19—C20	119.8 (2)
C6—C5—C4	113.0 (2)	C18—C19—C20	116.1 (2)
C1—C5—C4	104.4 (2)	C21—C20—C19	122.6 (3)
N2—C6—C7	120.4 (2)	C21—C20—Cl3	119.4 (2)
N2—C6—C5	112.2 (2)	C19—C20—Cl3	118.0 (2)
C7—C6—C5	102.5 (2)	C20—C21—C22	119.2 (3)
N2—C6—H6A	107.0	C20—C21—H21A	120.4
C7—C6—H6A	107.0	C22—C21—H21A	120.4
C5—C6—H6A	107.0	C23—C22—C21	121.0 (2)
C6—C7—C8	102.3 (2)	C23—C22—Cl4	119.6 (2)
C6—C7—H7A	111.3	C21—C22—Cl4	119.4 (2)
C8—C7—H7A	111.3	C22—C23—C18	119.8 (3)
C6—C7—H7B	111.3	C22—C23—H23A	120.1
C8—C7—H7B	111.3	C18—C23—H23A	120.1
			
O2—Ni1—O1—C12	−167.0 (2)	C6—C5—C9—C8	−37.3 (3)
N2—Ni1—O1—C12	−87.9 (4)	C1—C5—C9—C8	−159.1 (2)
N1—Ni1—O1—C12	26.2 (2)	C4—C5—C9—C8	82.4 (3)
O1—Ni1—O2—C19	172.9 (2)	C7—C8—C9—C5	12.0 (3)
N2—Ni1—O2—C19	8.3 (2)	C1—N1—C10—C11	−165.9 (3)
N1—Ni1—O2—C19	−116.8 (4)	Ni1—N1—C10—C11	4.6 (4)
O2—Ni1—N1—C10	−88.5 (4)	N1—C10—C11—C12	11.5 (4)
O1—Ni1—N1—C10	−19.3 (2)	N1—C10—C11—C16	−175.7 (3)
N2—Ni1—N1—C10	146.4 (2)	Ni1—O1—C12—C11	−18.5 (4)
O2—Ni1—N1—C1	81.9 (4)	Ni1—O1—C12—C13	162.3 (2)
O1—Ni1—N1—C1	151.08 (19)	C16—C11—C12—O1	−177.0 (3)
N2—Ni1—N1—C1	−43.26 (19)	C10—C11—C12—O1	−4.5 (4)
O2—Ni1—N2—C17	4.1 (2)	C16—C11—C12—C13	2.2 (4)
O1—Ni1—N2—C17	−73.4 (4)	C10—C11—C12—C13	174.7 (2)
N1—Ni1—N2—C17	172.7 (2)	O1—C12—C13—C14	177.7 (3)
O2—Ni1—N2—C6	174.05 (17)	C11—C12—C13—C14	−1.5 (4)
O1—Ni1—N2—C6	96.6 (4)	O1—C12—C13—Cl1	−2.0 (4)
N1—Ni1—N2—C6	−17.40 (18)	C11—C12—C13—Cl1	178.8 (2)
C10—N1—C1—C2	109.6 (3)	C12—C13—C14—C15	0.2 (5)
Ni1—N1—C1—C2	−61.6 (3)	Cl1—C13—C14—C15	179.9 (2)
C10—N1—C1—C5	−130.8 (2)	C13—C14—C15—C16	0.5 (5)
Ni1—N1—C1—C5	58.1 (3)	C13—C14—C15—Cl2	−178.8 (2)
N1—C1—C2—C3	154.6 (2)	C14—C15—C16—C11	0.2 (5)
C5—C1—C2—C3	31.1 (3)	Cl2—C15—C16—C11	179.5 (2)
C1—C2—C3—C4	−42.6 (3)	C12—C11—C16—C15	−1.6 (4)
C2—C3—C4—C5	38.5 (3)	C10—C11—C16—C15	−174.2 (3)
N1—C1—C5—C9	107.9 (3)	C6—N2—C17—C18	178.2 (2)
C2—C1—C5—C9	−129.7 (3)	Ni1—N2—C17—C18	−12.2 (4)
N1—C1—C5—C6	−6.2 (3)	N2—C17—C18—C23	−170.7 (2)
C2—C1—C5—C6	116.1 (3)	N2—C17—C18—C19	8.5 (4)
N1—C1—C5—C4	−129.7 (2)	Ni1—O2—C19—C18	−13.3 (4)
C2—C1—C5—C4	−7.4 (3)	Ni1—O2—C19—C20	169.04 (18)
C3—C4—C5—C9	105.1 (3)	C23—C18—C19—O2	−176.0 (2)
C3—C4—C5—C6	−143.2 (2)	C17—C18—C19—O2	4.8 (4)
C3—C4—C5—C1	−19.4 (3)	C23—C18—C19—C20	1.8 (4)
C17—N2—C6—C7	−1.1 (4)	C17—C18—C19—C20	−177.4 (2)
Ni1—N2—C6—C7	−171.80 (19)	O2—C19—C20—C21	176.2 (2)
C17—N2—C6—C5	−121.8 (2)	C18—C19—C20—C21	−1.7 (4)
Ni1—N2—C6—C5	67.5 (2)	O2—C19—C20—Cl3	−3.3 (3)
C9—C5—C6—N2	179.9 (2)	C18—C19—C20—Cl3	178.82 (19)
C1—C5—C6—N2	−57.2 (3)	C19—C20—C21—C22	0.7 (4)
C4—C5—C6—N2	61.4 (3)	Cl3—C20—C21—C22	−179.8 (2)
C9—C5—C6—C7	49.4 (3)	C20—C21—C22—C23	0.2 (4)
C1—C5—C6—C7	172.2 (2)	C20—C21—C22—Cl4	178.4 (2)
C4—C5—C6—C7	−69.2 (3)	C21—C22—C23—C18	−0.1 (4)
N2—C6—C7—C8	−167.4 (2)	Cl4—C22—C23—C18	−178.3 (2)
C5—C6—C7—C8	−42.0 (3)	C19—C18—C23—C22	−1.0 (4)
C6—C7—C8—C9	18.2 (3)	C17—C18—C23—C22	178.3 (2)
